# Cognitive Profiles of Children with Reading Disabilities and/or ADHD

**DOI:** 10.3390/bs16010012

**Published:** 2025-12-20

**Authors:** Miao Li, John R. Kirby, Tingzhao Wang, Wei Zhao

**Affiliations:** 1 College of Education, University of Houston, Houston, TX 77204, USA; 2 Faculty of Education, Queen’s University, Kingston, ON K7M 5R7, Canada; 3 Faculty of Education, Shaanxi Normal University, Xi’an 710062, China

**Keywords:** attention-deficit/hyperactivity disorder, cognitive profiles, comorbidity, reading disabilities

## Abstract

Building on prior work, this study examined cognitive profiles of children with reading disabilities (RD), attention-deficit/hyperactivity disorder (ADHD), and their comorbidity (ADHD + RD) compared to typically developing (TD) peers. Participants included 151 Grade 1–3 students, where there were 31 students with RD, 43 with ADHD, 27 with ADHD + RD, and 50 TD in China. Children were assessed in four cognitive domains: attention, inhibition, working memory, and rapid automatized naming (RAN), with age statistically controlled. Significant group differences emerged in each domain. The TD group consistently outperformed all groups. The comorbid ADHD + RD group showed pronounced deficits in attention, inhibition, and RAN. One-way ANCOVAs and multivariate analyses indicated that both RD and ADHD groups showed weaknesses in attention and RAN, with RD group weaker in working memory and ADHD group in inhibition. A 2 × 2 factorial ANCOVA confirmed significant main effects of RD and/or ADHD across domains, with no significant interaction effects, supporting an additive model. Findings highlight distinct and overlapping cognitive challenges associated with RD and ADHD and underscore the need for domain-specific intervention planning.

## 1. Introduction

Reading disabilities (RD) and attention-deficit/hyperactivity disorder (ADHD) are prevalent neurodevelopmental disorders that significantly impact academic performance and long-term developmental outcomes in school-aged children (American Psychiatric Association, 2013; Shaywitz & Shaywitz, 2020). RD, characterized by difficulties in word recognition, decoding, and reading fluency despite adequate intelligence and instruction, affects approximately 5–10% of children globally (Snowling et al., 2020). ADHD, defined by persistent inattention, impulsivity, and/or hyperactivity inappropriate for a child’s age, has a worldwide prevalence of 5–7% (Polanczyk et al., 2015). The frequent co-occurrence of RD and ADHD, with 20–40% of children with one condition also meeting criteria for the other, suggests shared cognitive and neurobiological mechanisms that may exacerbate challenges in the comorbid group (ADHD + RD) (Boada et al., 2012). Understanding whether cognitive deficits in ADHD + RD are additive, interactive, or unique compared to RD or ADHD alone is critical for designing effective interventions.

Prior research has identified deficits in key cognitive domains—attention, inhibition, working memory, and rapid automatized naming (RAN)—as central to RD and/or ADHD (Barkley, 2015; Kim et al., 2018; Pennington et al., 1993; Willcutt et al., 2001). Attention measures the ability to maintain focus on auditory stimuli while inhibition assesses the ability to suppress automatic responses (Barkley, 1997). Working memory involves the active, deliberate processing and manipulation of information temporarily stored in short-term memory (Baddeley, 2007). RAN refers to the ability to quickly and accurately naming a sequence of familiar visual stimuli, such as letters, digits, colors, or objects, which measures the speed and efficiency of retrieving well-known items (Wolf & Bowers, 1999). However, the extent to which these deficits are unique or overlapping across RD, ADHD, and their comorbidity remains debated. For example, RD is often linked to phonological processing and RAN deficits, which impair reading fluency (Kirby et al., 2025; Snowling & Hulme, 2022; Wolf & Bowers, 1999), while ADHD is associated with attention and inhibition (Barkley, 2015; Willcutt et al., 2010). The comorbid group may exhibit additive deficits, combining weaknesses from both conditions, or unique profiles that differ from either condition alone (McGrath et al., 2011; Pennington, 2006). Moreover, most research has focused on Western populations, with limited research on non-Western samples, such as children in China, where cultural and linguistic factors may influence cognitive performance. For example, the logographic nature of Chinese orthography, which relies heavily on morphological processing rather than grapheme-phoneme mapping, may uniquely affect cognitive deficits in RD and ADHD (Lee, 2024; Wei et al., 2025). The overall prevalence of ADHD among children and adolescents in China was 6.26%, which was generally consistent with the worldwide prevalence (T. Wang et al., 2017). The prevalence of RD among children and adolescence in China was 5%—12%, depending on different cutoff criteria, which was also similar to that of other countries (Cheng et al., 2021; Lin et al., 2020). These findings highlight the importance of cross-linguistic research to clarify the nature of cognitive deficits and inform targeted interventions for RD and ADHD in diverse populations.

Building on our prior work (Li et al., 2023), which investigated reading fluency in children with RD and/or ADHD and highlighted the predictive role of morphology in reading fluency in a Chinese sample, the present study shifted lens to the underlying cognitive mechanism of Chinese children aged 6–9 years (Grades 1–3) with RD, ADHD, ADHD + RD, and typically developing (TD) peers across four cognitive domains: attention, inhibition, working memory, and RAN. Using standardized assessments and controlling for age, we aimed to clarify the nature of cognitive deficits in the comorbid group and identify domain-specific challenges to inform intervention strategies, thereby addressing a gap in understanding cognitive profiles of children with RD and/or ADHD in a Chinese context. Throughout the paper, the terms ‘deficit’ and ‘impairment’ are used to refer to statistically significantly poorer performance and moderate-to-large effect sizes relative to the age-matched typically developing control group on the cognitive measures. These terms do not imply that every child met clinical criteria for impairment on the specific measure. It is important to note that, while group-level cognitive difficulties are commonly reported in ADHD, there is considerable heterogeneity: not all children with ADHD exhibit impairments in any given domain (Kofler et al., 2010). Furthermore, children with ADHD can demonstrate strengths, including creativity, high energy, and resilience (Sedgwick et al., 2019), which deserves recognition in research and practice.

### 1.1. Reading Disabilities and Cognitive Deficits

Reading disabilities (RD), are characterized by difficulties in phonological processing, decoding, and reading fluency, despite adequate intelligence and educational opportunities (Snowling & Hulme, 2022). A prominent cognitive model, the double-deficit hypothesis, posits that RD is associated with deficits in phonological awareness and RAN, with RAN reflecting the speed of accessing verbal labels for visual stimuli (Wolf & Bowers, 1999). In Chinese, a logographic language, RAN deficits are particularly robust predictors of RD due to demands on visual-orthographic processing (Liao et al., 2015; Peng et al., 2017; L.-C. Wang et al., 2018).

While phonological awareness and RAN are predominant in RD, RD also involves a multifaceted cognitive profile that extends to executive functions and memory systems (Kim et al., 2018; Vellutino et al., 2004). Working memory deficit is found to be prevalent in RD, as reading requires holding and manipulating phonological and orthographic information (Gathercole & Baddeley, 2014). For example, a meta-analysis by Kudo et al. (2015) highlighted that children with RD exhibit significant deficit/impairments in working memory, which correlates with poor reading comprehension.

Children with RD also exhibit deficits in attention and inhibition, which are executive function skills critical for reading development. These deficits may arise due to the cognitive demands of reading (e.g., decoding, phonological processing) or shared neurocognitive mechanisms with ADHD, given the high comorbidity rate (20–40%) between RD and ADHD (Boada et al., 2012). For example, Boada et al. (2012) reported that the attention and inhibition deficits are often less severe in RD than in ADHD but contribute to reading difficulties, especially in tasks requiring sustained focus for decoding. In a study of 104 Chinese children aged 6–12, Lee (2024) found that children with RD alone showed significant deficits in sustained attention (d = 0.45) and behavioral inhibition (d = 0.35) compared to typically developing TD peers. These deficits were linked to difficulties in word reading and fluency, as attention and inhibition support phonological processing and rapid naming. The RD-only group’s impairments were less severe than those in the ADHD + RD group, suggesting a distinct contribution of RD to these executive function deficits.

### 1.2. ADHD and Cognitive Deficits

Children with ADHD showed significant impairments in sustained attention and inhibitory control, compared to TD peers, with deficits persisting across developmental stages (Pievsky & McGrath, 2018). Attention deficits are particularly evident in tasks requiring resistance to distraction, where children with ADHD show increased errors of omission, slower reaction times, and greater response variability (Thomson et al., 2020). Similarly, children with ADHD also struggle to inhibit responses on tasks requiring them to name alternate shapes or opposite arrow directions, reflecting challenges in response suppression and cognitive flexibility (Schachar et al., 2000). The persistence of these deficits across developmental stages is well-documented (Berlin et al., 2004; Pang et al., 2025; Thomson et al., 2020). Longitudinal studies indicate that while some children with ADHD show partial improvement in sustained attention and inhibitory control during adolescence, significant impairments remain relative to TD peers, particularly in those with persistent ADHD diagnoses (Qian et al., 2013; Pang et al., 2025; Thomson et al., 2020). For example, Qian et al. (2013) found that Chinese children and adolescents with ADHD displayed delayed developmental trajectories on inhibition compared to controls of the same age.

Working memory impairments are also well-documented in ADHD, especially in the inattentive subtype (Kofler et al., 2008; Martinussen et al., 2005). Working memory is impaired in 68–85% of children with ADHD (Karalunas et al., 2017; Kasper et al., 2012). For example, Ramos et al. (2020) conducted a meta-analysis study and reported that children and adolescents with ADHD exhibited significant impairments in working memory, with a large effect size (Hedges’ g = 0.76, *p* < 0.001), indicating poorer performance compared to TD peers. While RAN is less consistently impaired in ADHD, some studies suggest slower naming speed due to attentional lapses, particularly in tasks requiring rapid processing (Tannock et al., 2000; E. Wang et al., 2017). For example, E. Wang et al. (2017) examined Chinese children with ADHD and found that attentional selection (the ability to focus on relevant stimuli while ignoring distractors) significantly predicted RAN performance, supporting the role of attentional impairments in slower naming speed.

### 1.3. Comorbidity of RD and ADHD

The high comorbidity rate of RD and ADHD (20–40%) suggests shared cognitive and neurobiological mechanisms (Boada et al., 2012). The comorbidity of RD and ADHD is often explained by two theoretical models: the additive model and the common etiology model. The additive model posits that children with ADHD + RD exhibit more severe deficits in attention, inhibition, working memory, and RAN due to the combined impairments of both disorders (Shanahan et al., 2006; Crisci et al., 2021; Lee, 2024). For example, Poon et al. (2021) found that Chinese children with RD, either alone or combined with ADHD, performed worse than those without RD on verbal and visual working memory. Children with RD + ADHD performed worse than those with single deficits, who performed worse than typically developing children, on teachers’ ratings of classroom behavior related to working memory. Lee (2024) found that Chinese children with ADHD + RD showed significantly greater deficits in verbal and visuospatial working memory (d = 0.92–1.10), behavioral inhibition (d = 0.98), and RAN tasks (d = 0.80) compared to single-diagnosis groups, reflecting the cumulative impact of ADHD-related executive function deficits and RD-related phonological and naming speed impairments. The additive model was also supported by L. C. Wang and Chung (2018) by reporting that Chinese children with both RD and ADHD exhibited more severe impairments in auditory working memory and RAN compared to children with only RD or ADHD.

Conversely, the common etiology model suggests that shared deficits, such as processing speed, underlie both conditions, leading to overlapping cognitive profiles (Germanò et al., 2010; Boada et al., 2012; DuPaul et al., 2013; McGrath et al., 2011). For instance, a review of comorbidity between RD and ADHD conducted by Sexton et al. (2012) highlighted that children with both conditions show decreased performance on reading-related tasks (e.g., word reading accuracy and fluency, phonological awareness, RAN, etc.) compared to typically developing peers, with no differences due to RD or ADHD alone in some areas (Sexton et al., 2012). And Boada et al. (2012) reported that ADHD + RD profiles resembled dyslexia-only impairments in attention and inhibition, driven by shared neurocognitive deficits. These mixed findings highlight the need for studies to test additive versus common etiology models.

### 1.4. The Present Study

The majority of research on RD and ADHD centers on Western populations, with less evidence from non-Western settings like China. Recent studies have explored cognitive profiles in Chinese populations, finding that inhibition, working memory, and RAN, are more pronounced in children with RD and ADHD compared to those with RD or ADHD alone, supporting the additive model (Lee, 2024; L. C. Wang & Chung, 2018; Wei et al., 2025). Some studies also support the common etiology model. For example, in their mediation analysis, Wu et al. (2024) showed that sustained attention and IQ explain the reading impairments in ADHD, suggesting that these cognitive deficits are shared mechanisms underlying both ADHD and RD. This aligns with the common etiology model, which posits that overlapping cognitive or neurobiological factors (e.g., attention, executive functions) drive comorbidity, leading to similar deficits in ADHD and RD groups.

Recent research suggests that cognitive profiles and their contributions to RD in Chinese children with RD, ADHD, or both are generally consistent with those observed in children using alphabetic languages, though further studies are needed to confirm these patterns. For instance, Lee (2024) found that Chinese children with ADHD + RD exhibited more pronounced deficits in visuospatial working memory (d = 0.92–1.10) compared to those with RD or ADHD alone, a pattern that differs from findings in alphabetic language populations, in which auditory working memory deficits are typically more pronounced in children with RD and ADHD. These results highlight the need for additional research to clarify cross-linguistic differences in cognitive profiles and their implications for the additive and common etiology models of RD-ADHD comorbidity.

The present study examined the cognitive profiles in attention, inhibition, working memory, and RAN, using a 2 × 2 factorial design to test additive versus interactive effects in a Chinese sample. To our knowledge, this is one of the first to systematically test the additive versus interactive models of comorbidity in a non-alphabetic, logographic writing system. The finding strengthens the cross-linguistic generalizability of the multiple-deficit model. Additionally, this study contributes to understanding domain-specific cognitive difficulties and informs culturally sensitive interventions. Current evidence-based programs developed in the West (e.g., phonological-training-heavy approach) may be less effective in Chinese without explicit components addressing unique features of Chinese. Our study would provide an empirical foundation for designing and prioritizing culturally and linguistically tailored protocols. The research questions are:

(1) Do children with ADHD, RD, ADHD + RD, and TD differ in their overall cognitive profiles across multiple domains?
(2) Do children with ADHD, RD, ADHD + RD, and TD show different patterns of performance across cognitive domains?
(3) Do ADHD and RD, alone or in combination, uniquely affect cognitive domains, and are comorbid effects additive or interactive?

We hypothesized that (1) children with neurodevelopmental disorders would show significantly poorer overall performance than TD children across most or all domains; (2) the four groups would display different profiles; and (3) both ADHD and RD groups would show domain-specific cognitive weaknesses and the comorbid group would show additive effects, based on the findings from the previous research (e.g., Shanahan et al., 2006; Crisci et al., 2021; Lee, 2024).

## 2. Methods

### 2.1. Participants

The study included 151 children in Grades 1–3 (mean age = 102.63 months) from a metropolitan city in Mainland China. The age range was 83–131. Participants were drawn from two sources: 81 children recruited from two elementary schools in lower- to middle-income neighborhoods and 70 children with ADHD diagnoses recruited from a local Children’s Hospital. There were 29 girls and 21 boys in the TD group, 11 girls and 20 boys in the RD group, 13 girls and 30 boys in the ADHD group, and 2 girls and 25 boys in the RD + ADHD group.

For the school-based sample, teachers identified children with either typical reading skills or reading difficulties, yielding 48, 53, and 49 children in Grades 1, 2, and 3, respectively. Teachers identified TD and RD children using a nomination procedure based on observed classroom reading performance (e.g., fluency, accuracy, and effort when reading grade-level material). These children completed a Chinese word reading accuracy screening task, with 25th percentile cutoffs of 44, 58, and 69 characters read correctly for Grades 1, 2, and 3, respectively. Children scoring below the 25th percentile were classified as RD (*n* = 31), while 50 TD children were randomly selected from those scoring above this threshold. This cutoff, though an artificial dichotomy, aligns with common practices in Chinese reading research due to the lack of standardized RD diagnostics (Cao et al., 2017; McBride et al., 2018; Peng et al., 2017). According to school records, the children did not have any history of diagnosed or suspected significant cognitive delays, significant behavioral problems, or frequent school absences, history of serious emotional/psychiatric disturbances (i.e., major depression, psychotic or pervasive developmental disorder, autism) or chronic neurologic conditions (i.e., seizure disorder, developmental neurological conditions, substance/teratogen exposure, Tourette or other tic disorders, acquired brain injuries), or documented vision or hearing impairment.

For the hospital-based sample, 70 children were diagnosed with ADHD by pediatric specialists using the Swanson, Nolan, and Pelham Rating Scale (SNAP-IV; Swanson et al., 2001) and DSM-5 criteria (American Psychiatric Association, 2013). These children were further assessed with the same word reading accuracy measure, identifying 43 with ADHD only and 27 with comorbid ADHD + RD (below 25th percentile). They were not screened for additional co-occurrences. All 70 children in the ADHD and ADHD + RD groups were medication-naïve at the time of testing. None had ever received stimulant or non-stimulant medication for ADHD prior to or during participation in the study. This information was confirmed by parental report and verified through medical records at the recruiting Children’s Hospital.

All participants attended schools following China’s standardized national curriculum, ensuring comparable instructional exposure. Parents reported annual household incomes primarily between RMB 50,000 and RMB 100,000 (US$7000–$14,000), indicative of lower- to middle-class status. Informed consent was obtained from parents, and the study was approved by the university’s Institutional Review Board. Children provided assent prior to the start of the testing.

### 2.2. Measures

#### 2.2.1. Word Reading Accuracy (Screening Measure)

To evaluate word reading accuracy and identify children with RD, a task developed by Zhang et al. (2012) was administered. Participants were presented with 150 single Chinese characters drawn from textbooks used in Grades 1–6. Children read each character aloud, and the task was stopped after 10 consecutive incorrect responses. The score reflected the total number of characters correctly read. The split-half reliability of the current sample was 0.98.

#### 2.2.2. Attention

Auditory Attention which is a subtest of NEPSY-II (A Developmental Neuropsychological Assessment, Korkman et al., 2007) was used to assess selective and sustained auditory attention, as well as the ability to resist distraction. The task requires the child to listen to a series of 180 words presented via an audio recording and respond only to a specific target word (e.g., “red”) by performing an action (e.g., placing a marker in a box) while ignoring all other words. The total score is the number of correct responses. The split-half reliability of the current sample was 0.82.

#### 2.2.3. Inhibition

The Inhibition subtest of NEPSY-II assesses inhibitory control and cognitive flexibility. It consists of three conditions: Naming, Inhibition, and Switching, administered with two sets of stimuli—Shapes and Arrows. Below are descriptions for each condition within the Shape and Arrow tasks.

**Shape Naming.** It is the baseline condition of the Inhibition subtest, assessing basic processing speed and naming ability. The child is presented with a series of 40 shapes (e.g., circles, squares) in different colors and asked to name each shape as quickly and accurately as possible. The total score is the completion time. The split-half reliability of the current sample was 0.81.

**Arrow Naming.** It is analogous to Shape Naming but uses arrows pointing in different directions (e.g., up, down, left, right) as stimuli. The child views a grid of 40 arrows and names the direction of each arrow as quickly and accurately as possible. The total score is the completion time. The split-half reliability of the current sample was 0.85.

**Shape Inhibitory.** This assesses inhibitory control by requiring the child to suppress an automatic response and provide an alternative response. The child must name the opposite of the shape they see (e.g., say “circle” when seeing a square, and vice versa). The child is shown the same grid of shapes as in Shape Naming but is instructed to name the opposite shape for each item (e.g., say “square” for a circle). The total score is the completion time. The split-half reliability of the current sample was 0.83.

**Arrow Inhibitory.** This is similar to Shape Inhibitory but uses arrows as stimuli. The child must name the opposite direction of each arrow (e.g., say “up” when the arrow points down). The total score is the completion time. The split-half reliability of the current sample was 0.80.

#### 2.2.4. Working Memory

The Digit Backward Span subtest of Wechsler Intelligence Scale for Children (WISC) was used to assess working memory by requiring the child to repeat a sequence of numbers in reverse order after hearing them presented orally by the examiner. The examiner reads a sequence of digits (e.g., “4-7-2”) at a rate of one digit per second. The child is instructed to repeat the sequence in reverse order (e.g., “2-7-4”). The sequences start with two digits and increase in length as the child progresses successfully. The task includes multiple trials, with two trials per sequence length, and continues until the child fails both trials of a given length or completes the longest sequence. The total score is the number of correct trials. The split-half reliability of the current sample was 0.76.

#### 2.2.5. Rapid Automatized Naming (RAN)

**RAN Digit Naming.** The Rapid Automatized Naming (RAN) Digits subtest from the Comprehensive Tests of Phonological Processing (Wagner et al., 1999) was administered to assess children’s digit naming speed. Children were instructed to name a sequence of digits (e.g., 2, 3, 4, 5, 7, 8), presented in a random order across four rows of nine items each, as quickly and accurately as possible. The efficiency score was calculated by dividing the number of correctly named digits by the total time taken to complete the task. The split-half reliability of the current sample was 0.83.

**RAN Color Naming.** The Rapid Automatized Naming (RAN) Color subtest from the Comprehensive Tests of Phonological Processing (Wagner et al., 1999) was administered to assess children’s color naming speed. Participants named a sequence of colored shapes (e.g., red, blue, yellow, green, black) arranged in four rows of nine items each, presented in random order, as quickly and accurately as possible. The efficiency score was computed by dividing the number of correctly named colors by the total time required to complete the task. The split-half reliability of the current sample was 0.79.

### 2.3. Procedure

Children were tested individually in a quiet room at their school (school-based sample) or at the Children’s Hospital (hospital-based sample). Testing was conducted by trained graduate students in special education. Each child completed the cognitive battery in a single session lasting approximately 60 min. Breaks were provided as needed.

### 2.4. Statistical Analysis

Power analysis was conducted using G*Power 3.1. For the mixed ANCOVA, assuming a medium effect size (f = 0.25), α = 0.05, power = 0.90, four groups, four repeated measures, and a correlation among repeated measures of 0.50, the required total sample size was 92. For the 2 × 2 factorial ANCOVAs per domain, the same parameters required N = 128.

To address research question 1, a series of analyses of covariance (ANCOVA) and a series of multivariate analyses of covariance (MANCOVAs) were employed to test whether the cognitive profiles differ overall across the four groups. ANCOVA with age as a covariate was conducted for attention and working memory, respectively. MANCOVAs with age as a covariate were carried out for inhibition and RAN, respectively, because each construct was assessed by more than one measure. Age was controlled because there was a wide range of participants (Grade 1–Grade 3). To address research question 2, a mixed-design ANCOVA which is a type of repeated measures ANCOVA was used with the cognitive domain as the within-subject factor and four groups as the between-subject factor to compare the patterns of performance across multiple cognitive domains across groups. Repeated measures are typically used when the same subjects are measured multiple times, but it can also be used when each subject provides multiple scores across a set of conceptually related dependent variables as is the case with cognitive domains in this study. To address research question 3, a series of 2 × 2 factorial ANCOVAs with ADHD status and RD status as independent variables was employed to examine which cognitive domains were affected by ADHD alone or RD alone and whether the combined effects in the comorbid ADHD + RD group were additive or interactive.

## 3. Results

Missing data patterns were analyzed and revealed that data were missing at random, as indicated by Little’s MCAR Test (χ^2^ = 27.02, df = 21, *p* = 0.17). To address missing values, multiple imputation was employed to ensure robust statistical analyses. Descriptive statistics, including means and standard deviations for age and performance on the word reading accuracy screening task, Attention, Inhibition, Working Memory, and RAN across the four groups are reported in Table 1. Table 2 presents correlations among all measures, with age-corrected correlations displayed above the diagonal to account for age effects in the cognitive profile analyses.

### 3.1. Reading Ability

A one-way ANCOVA, with age as a covariate, was performed to compare word reading accuracy (screening measure) across the four groups (see Table 1). Significant between-group differences emerged, *F*(3, 145) = 72.37, *p* < 0.001. Bonferroni-corrected post hoc tests (*p* < 0.05) revealed that the TD group outperformed the RD, ADHD, and ADHD + RD groups. The ADHD group also scored higher than the RD group, while the ADHD + RD group’s performance was comparable to the RD group but lower than the ADHD group.

### 3.2. Comparison of Cognitive Profiles Across the Four Groups

In order to address research question 1 on whether the cognitive profiles differ overall across the four groups, a series of ANCOVAs and MANCOVAs was conducted on attention, inhibition, working memory, and RAN.

#### 3.2.1. Attention

A one-way ANCOVA was conducted to compare performance on attention across the four groups after age was controlled. Between-group differences were statistically significant, *F*(3, 145) = 12.63, *p* < 0.001. Post hoc tests with Bonferroni correction (*p* < 0.01) showed that the TD group had significantly higher scores than the other three groups. The ADHD group performed similarly to the RD group. The ADHD + RD group performed worse than the other three groups.

#### 3.2.2. Inhibition

There was a statistically significant multivariate effect on the measures of inhibition after controlling for age, Wilks’ λ = 0.63, *F*(12, 376) = 6.17, *p* < 0.001. Follow-up univariate one-way ANCOVAs indicated significant group differences in Shape Naming, *F*(3, 145) = 8.83, *p* < 0.001, η_p_^2^ = 0.15, Arrow Naming, *F*(3, 145) = 19.20, *p* < 0.001, η_p_^2^ = 0.28, Shape Inhibitory, *F*(3, 145) = 12.33, *p* < 0.001, η_p_^2^ = 0.20, and Arrow Inhibitory, *F*(3, 145) = 10.32, *p* < 0.001, η_p_^2^ = 0.17. Post hoc Bonferroni *t*-tests (*p* < 0.01) confirmed that the TD group had significantly higher scores than the RD, ADHD, and ADHD + RD groups on all inhibition measures except the Shape Naming task. The ADHD + RD group performed worse than the other three groups on the Arrow Naming task. The ADHD + RD group performed similarly to the RD group or the ADHD group on tasks of Shape Naming, Shape Inhibitory, and Arrow Inhibitory. There was no significant difference between the RD and ADHD groups.

#### 3.2.3. Working Memory

A one-way ANCOVA was conducted to compare performance on working memory across the four groups after age was controlled. Between-group differences were statistically significant, *F*(3, 145) = 4.31, *p* < 0.01. Post hoc tests with Bonferroni correction (*p* < 0.05) showed that the TD group had significantly higher scores than the RD group. There was no significant difference among the RD, ADHD, and ADHD + RD groups.

#### 3.2.4. RAN

There was a statistically significant multivariate effect on the measures of RAN Digit Naming and Color Naming efficiency after controlling for age, Wilks’ λ = 0.59, *F*(6, 288) = 14.69, *p* < 0.001. Follow-up univariate one-way ANCOVAs indicated significant group differences in Digit Naming, *F*(3, 145) = 26.19, *p* < 0.001, η_p_^2^ = 0.35 and Color Naming, *F*(3, 145) = 17.60, *p* < 0.001, η_p_^2^ = 0.26. Post hoc Bonferroni *t*-tests (*p* < 0.01) confirmed that the TD group had significantly higher scores than the RD, ADHD, and ADHD + RD groups on both RAN Digit Naming and Color Naming. The ADHD group performed similarly to the RD group on both RAN Digit Naming and Color Naming. The ADHD + RD group performed worse than the other three groups on Digit Naming. However, the ADHD + RD group performed similarly to the RD and ADHD groups on Color Naming, all three being worse than the TD group.

### 3.3. Patterns of Performance of Cognitive Profiles Across Groups

In order to address research question 2 and show how patterns differ across four cognitive domains and four groups, a mixed ANCOVA was used with age as the covariate. Because Inhibition and RAN have more than one task to be measured, we averaged the z-scores of the four tasks of Inhibition and two tasks of RAN. The inhibition scores were also reversed so that higher scores reflected better performance. Specifically, raw completion times (in seconds) for the Inhibition task, where lower times indicate stronger inhibitory control, were transformed by multiplying by −1 (i.e., negative time scores). Then we standardized each cognitive domain score to ensure all measures were on a common scale. A 4 (Cognitive Domain: Attention, Inhibition, Working Memory, RAN) x 4 (Group: TD, RD, ADHD, ADHD + RD) mixed-design repeated measures ANCOVA with age controlled revealed a significant main effect of cognitive domain, *F*(3, 435) = 3.33, *p* < 0.05, η_p_^2^ = 0.02, a significant main effect of group, *F*(3, 145) = 8.71, *p* < 0.001, η_p_^2^ = 0.15, and a significant interaction effect, *F*(9, 435) = 9.22, *p* < 0.001, η_p_^2^ = 0.16. The pairwise comparisons of the cognitive domains did not yield statistically significant differences between individual domains (all *p*s > 0.05). The pairwise comparisons of the groups showed that the TD group performed the best among all four groups. The ADHD + RD group performed worse than the TD group and the ADHD group. For the Domain x Group interaction effect, the pairwise comparisons indicated that the TD group did well in all domains. In attention, the ADHD group performed similarly to the RD group, and the ADHD + RD group performed worse than the other three groups. In Inhibition, the ADHD + RD group performed significantly worse than the RD group, and there were no significant differences between the RD group and the ADHD group, or between the ADHD + RD group and the ADHD group. In Working Memory, there was no significant difference among the RD, ADHD, and ADHD + RD groups. In RAN, the ADHD group performed similarly to the RD group, and the ADHD + RD group performed worse than the other three groups.

**Figure 1:**
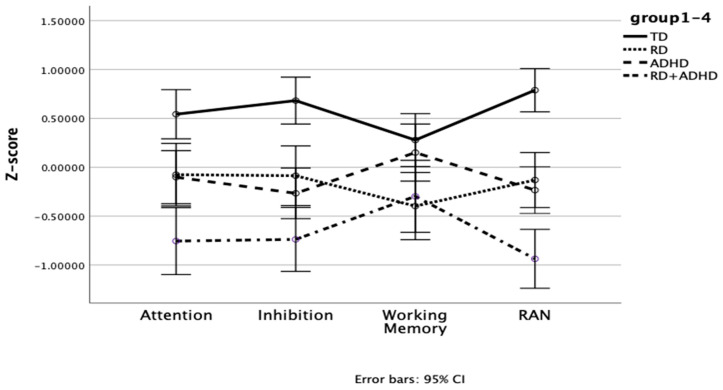
The age-corrected cognitive profiles across the four groups.

To better illustrate the results, a visual representation of the cognitive profiles across the four groups is shown in Figure 1, as age-corrected means. The Figure illustrates how the cognitive profiles differ among groups. The TD group’s profile is consistently high across all domains, reflecting strengths in all cognitive areas; only in the domain of working memory is it close to one of the three other groups, to the ADHD group. In contrast, the ADHD + RD group scores consistently poorly, except in the domain of Working Memory, in which is equivalent to the RD group. The two single deficit groups, ADHD group and RD group, perform similarly except in the domain of Working Memory, in which the ADHD group does better. The key difference for explaining the interaction is that Working Memory is related to reading: the two groups who score highly on reading (TD and ADHD) do better on Working Memory than the two groups who have reading difficulties (RD and ADHD + RD). This plot supports the idea that cognitive strengths and weaknesses differ among the groups, not just in overall level but also in pattern.

### 3.4. Interactive Versus Additive Effects of ADHD and RD on Cognitive Domains

In order to address research question 3 on additive or interaction effect, a series of 2 (ADHD status: present vs. absent) × 2 (RD status: present vs. absent) factorial ANCOVA was conducted to examine the additive or interactive effects of ADHD and RD on the four cognitive domains, controlling for age; these effects are depicted in Figure 1. For attention, there was a significant main effect of ADHD status, *F*(1, 145) = 15.60, *p* < 0.001, partial η^2^ = 0.10, indicating that children with ADHD scored lower on attention than those without ADHD. The main effect of RD status was also significant, *F*(1, 145) = 9.64, *p* < 0.01, partial η^2^ = 0.06. However, the interaction between ADHD and RD was not significant. For inhibition, a significant main effect of ADHD status was found, *F*(1, 145) = 24.10, *p* < 0.001, partial η^2^ = 0.14, indicating that children with ADHD scored lower on inhibition than those without ADHD. However, the main effect of RD status and the interaction effect between ADHD and RD were not significant. For working memory, a significant main effect of RD status was found, *F*(1, 145) = 8.97, *p* < 0.01, partial η^2^ = 0.06, indicating that children with RD scored lower on working memory than those without RD. However, the main effect of ADHD status and the interaction effect between ADHD and RD were not significant. For RAN, there was a significant main effect of ADHD status, *F*(1, 145) = 37.80, *p* < 0.001, partial η^2^ = 0.21, showing that children with ADHD scored lower on RAN than those without ADHD. The main effect of RD status was also significant, *F*(1, 145) = 17.91, *p* < 0.01, partial η^2^ = 0.11, indicating that children with RD scored lower on RAN than those without RD. However, the interaction between ADHD and RD was not significant. These results, with main effects but no interactions, indicate that ADHD and RD independently contribute to weaknesses in attention and RAN (additive effects), while inhibition deficits are specific to ADHD, and working memory deficits are specific to RD.

## 4. Discussion

The present study investigated cognitive profiles in attention, inhibition, working memory, and RAN among Chinese children with RD, ADHD, ADHD + RD, and TD peers. The findings addressed the three research questions by revealing significant group differences in overall cognitive profiles, distinct patterns of performance across domains, and unique but additive effects of RD and ADHD on these domains. These results provided support for the additive model of comorbidity and offered insights into domain-specific difficulties, with implications for interventions in Chinese children.

### 4.1. Group Differences in Overall Cognitive Profiles

Consistent with prior research (Barkley, 2015; Snowling & Hulme, 2022), significant group differences emerged in each domain, with the TD group consistently outperforming the clinical groups. The ADHD + RD group exhibited the most pronounced poor performance, particularly in attention and RAN. These findings align with the literature, where comorbid ADHD + RD is associated with compounded cognitive challenges (Lee, 2024; Poon et al., 2021). For example, the ADHD + RD group’s poorer performance on attention (lower than all groups) and RAN (worse than all groups on Digit Naming) suggests that comorbidity exacerbates difficulties beyond single-diagnosis groups, supporting the notion that ADHD + RD represents a more severe cognitive burden (Shanahan et al., 2006).

### 4.2. Patterns of Performance Across Cognitive Domains

The mixed ANCOVA revealed a significant interaction effect, indicating that cognitive strengths and weaknesses varied among groups. The TD group performed well across all domains, while the ADHD + RD group was particularly poor in attention, inhibition, and RAN. In contrast, the RD and ADHD groups showed similar patterns in attention and RAN, with no significant differences between them, but domain-specific weaknesses: inhibition difficulties were prominent in ADHD, and working memory difficulties were evident in RD. Our working memory task was backwards digit span, similar to the verbal working memory task used by Poon et al. (2021). Like Poon et al., we found that RD was linked to poor verbal working memory. Children with RD, who typically exhibit phonological processing weaknesses, may therefore show greater difficulty on this task, especially in a logographic orthography where verbal rehearsal is critical for character recognition (Liao et al., 2015; Peng et al., 2017). While research shows that ADHD group has deficits in working memory (e.g., Lee, 2024; Ramos et al., 2020), it is also reported that children with ADHD exhibited larger deficits in spatial working memory than in verbal working memory (Martinussen et al., 2005). Our study did not administer a spatial working memory task, or the teacher rating scales that Poon et al. (2021) employed, which may explain why we did not observe a significant working memory difficulty in the ADHD-only group. Figure 1 illustrates the distinct group profiles, highlighting how the “shape” of difficulties differs, with ADHD + RD showing a more flattened profile indicative of broader difficulties.

### 4.3. Interactive Versus Additive Effects of ADHD and RD

The 2 × 2 factorial ANCOVA was intended to assess the question of interactive versus additive effects. It revealed significant main effects of ADHD and RD on attention and RAN, with no significant interaction effects. Inhibition was uniquely affected by ADHD (*F*(1, 145) = 24.10, *p* < 0.001, η^2^ = 0.14), and working memory by RD (*F*(1, 145) = 8.97, *p* < 0.01, η^2^ = 0.06). These findings confirm the additive model, as RD and ADHD independently contribute to poor performance, with the ADHD + RD group experiencing cumulative struggles, particularly in attention and RAN (Pennington, 2006; Lee, 2024). For example, L. C. Wang and Chung (2018) reported more severe deficits in auditory working memory and RAN in Chinese children with ADHD + RD, mirroring our results. The absence of interaction effects indicates that the comorbid group’s difficulties are a summation of RD and ADHD poor performance. The non-significant interactions confirm that ADHD + RD’s performance is explained by the independent effects of ADHD and RD (e.g., ADHD affects Inhibition, RD affects Working Memory), supporting the additive model.

Regarding the differing interaction results, the mixed ANCOVA (to address RQ2) and factorial ANCOVA (to address RQ3) answer different questions about the same data, and their different interaction results reflect their distinct designs and grouping structures. The mixed ANCOVA’s interaction reflects group-specific profile differences across domains, while the factorial ANCOVA’s lack of interactions indicates that ADHD and RD effects are additive within each domain. Both support the additive model but from different angles.

### 4.4. Theoretical and Practical Implications

The robust support for the additive model aligns with prior Chinese studies, such as Lee (2024), which found greater deficits in visuospatial working memory and RAN in ADHD + RD compared to single-diagnosis groups, and Poon et al. (2021), which reported additive deficits in teacher-rated working memory behavior. The findings extend the literature by demonstrating that, in Chinese children, the logographic nature of the language amplifies RAN difficulties in RD and ADHD + RD due to visual-orthographic demands (Liao et al., 2015; Peng et al., 2017). The domain-specific effects (inhibition in ADHD, working memory in RD) highlight the distinct contributions of each disorder, reinforcing the additive model’s applicability in non-Western contexts. These results address a gap in the predominantly Western-focused literature (Boada et al., 2012) and underscore the importance of cross-linguistic research, given the 6.26% ADHD prevalence in China (T. Wang et al., 2017).

The additive difficulties in ADHD + RD, particularly in attention and RAN, suggest the need for multi-component interventions tailored to Chinese children. For RD, phonological and RAN training could enhance reading fluency, addressing visual-orthographic challenges (Snowling & Hulme, 2022). For ADHD, attention and inhibition-focused interventions, such as cognitive-behavioral therapy or Go/No-Go training (Schachar et al., 2000), are warranted. The ADHD + RD group would benefit from combined approaches, integrating phonological exercises with attention training (e.g., Klingberg et al., 2005). Teachers and schools in China should develop tiered, individualized education plans that account for these domain-specific profiles, particularly in high-stakes academic environments where reading difficulties impact performance. At the policy level, the results underscore the need for educational authorities to move toward a multiple-deficit framework that explicitly includes executive-function and rapid naming components. Updating national guidelines and teacher-training curricula would improve early identification and support for the substantial proportion of children affected by ADHD, RD, or both.

### 4.5. Limitations and Future Directions

Limitations include the modest sample size (N = 151, with *n* = 27 for ADHD + RD), which may limit power to detect subtle interaction effects. Future studies with larger samples could further validate additive effects. The cross-sectional design restricts inferences about developmental trajectories. Future longitudinal studies could track how these deficits evolve, as in Qian et al. (2013). A further limitation is the absence of data on children’s sleep quality and sleep disorders. Sleep disturbances are common in ADHD and have been shown to affect procedural learning (Kirov & Brand, 2014). Future studies should incorporate validated instruments such as the Sleep Disturbance Scale for Children (Bruni et al., 1996) to examine whether sleep affects the cognitive deficits observed in the ADHD group and/or comorbid groups. Additionally, most cognitive domains were assessed using a single test, which may limit construct validity by not fully capturing the multifaceted nature of these abilities. Future research should incorporate multiple measures per domain to enhance validity. Finally, an important next step will be to examine the relationship between cognitive profiles and both the types and severity of ADHD behaviors. Given the dimensional nature of ADHD and evidence of developmental changes in symptom expression (Hudziak et al., 2007; Heidbreder, 2015), future studies should incorporate measures of symptom severity and test whether certain ADHD symptoms may be more strongly linked to some specific cognitive difficulties. Such analyses would further refine the additive model and inform symptom-specific intervention targets.

## 5. Conclusions

This study provides strong evidence for the additive model of RD-ADHD comorbidity in Chinese children, with the ADHD + RD group showing the most severe difficulties in attention and RAN. Domain-specific findings (inhibition in ADHD, working memory in RD) inform targeted interventions, addressing a critical gap in non-Western research. By highlighting the compounded cognitive challenges in ADHD + RD, this study supports the development of culturally sensitive interventions to improve academic outcomes in Chinese children with these neurodevelopmental disorders.

## Figures and Tables

**Table 1:** Descriptive Statistics, ANCOVAs, and Multiple Comparisons for the Four Groups.

Measures	TD M (SD)	RD M (SD)	ADHD M (SD)	ADHD + RD M (SD)	Comparison ^a^
Age Attention	106.46 (9.79)	101.77 (11.04)	100.64 (10.09)	101.65 (9.39)	TD = R = A = R + A
Auditory Attention	28.04 (2.81)	25.90 (2.99)	25.81 (2.30)	23.56 (4.58)	TD > R = A > R + A
Inhibition					
Shape Naming	24.47 (4.50)	26.52 (4.99)	28.28 (6.60)	31.60 (8.21)	TD > R = A > R + A
Arrow Naming	23.67 (5.64)	28.06 (5.05)	28.31 (5.76)	34.21 (7.13)	TD > R = A > R + A
Shape Inhibitory	32.56 (6.30)	40.06 (7.98)	40.87 (9.78)	42.30 (8.50)	TD > R = A = R + A
Arrow Inhibitory	38.44 (9.14)	45.39 (9.80)	47.46 (9.49)	49.62 (11.12)	TD > R = A = R + A
Working Memory					
Digit Backward Span	6.98 (2.35)	5.61 (1.38)	6.72 (1.91)	5.82 (1.80)	TD > R = A = R + A
RAN					
RAN Digit	2.42 (0.57)	1.88 (0.43)	1.92 (0.49)	1.43 (0.28)	TD > R = A > R + A
RAN Color	1.20 (0.34)	0.99 (0.22)	0.87 (0.24)	0.78 (0.24)	TD > R = A = R + A
Word Reading Accuracy	113.26 (13.28)	55.97 (11.69)	85.07 (23.29)	42.70 (13.72)	TD > A > R = R + A

*Note*. R = RD; A = ADHD; R + A = ADHD + RD. ^a^ In these comparisons, an equal sign indicates nonsignificant difference, and the less-than sign indicates *p* < 0.05.

**Table 2:** Correlations of All Measures.

Measures	1	2	3	4	5	6	7	8	9
1. Attention	1	−0.21 *	−0.37 **	−0.27 **	−0.23 **	0.11	0.31 **	0.29 **	0.25 **
2. Arrow Naming	0.43 **	1	0.46 **	0.41 **	0.39 **	−0.00	−0.31 **	−0.36 **	−0.18 *
3. Shape Naming	−0.28 **	0.53 **	1	0.42 **	0.62 **	−0.16 *	−0.49 **	−0.39 **	−0.52 **
4. Shape Inhibitory	−0.33 **	0.49 **	0.48 **	1	0.65 **	−0.16	−0.32 **	−0.33 **	−0.34 **
5. Arrow Inhibitory	−0.29 **	0.67 **	0.46 **	0.67 **	1	−0.16	−0.41 **	−0.33 **	−0.38 **
6. Working Memory	0.13	−0.20 *	−0.06	−0.20 *	−0.20 *	1	0.15	0.33 **	0.18 *
7. RAN Digit	0.39 **	−0.55 **	−0.41 **	−0.39 **	−0.48 **	0.18 *	1	0.53 **	0.46 **
8. RAN Color	0.34 **	−0.45 **	−0.42 **	−0.38 **	−0.38 **	0.35 **	0.57 **	1	0.37 **
9. Word reading accuracy	0.37 **	−0.60 **	−0.37 **	−0.44 **	−0.48 **	0.23 **	0.58 **	0.44 **	1

*Note*. N = 151; Age corrected (above diagonal) and uncorrected (below diagonal). * *p* < 0.05, ** *p* < 0.001.

## Data Availability

The raw data supporting the conclusions of this article will be made available by the authors on request.
